# Self-medication Practices among the Peri-urban Households of Two Communities of Dharan Sub-metropolitan city of Eastern Nepal: A Descriptive Cross-sectional Study

**DOI:** 10.31729/jnma.5185

**Published:** 2020-08-31

**Authors:** Kumud Chapagain, Gajendra Prasad Rauniyar

**Affiliations:** 1 Department of Clinical Pharmacology & Therapeutics, B.P. Koirala Institute of Health Sciences, Dharan, Nepal

**Keywords:** *Nepal*, *prevalence*, *self-medication*

## Abstract

**Introduction:**

Self-medication practice is the use of medication without prescription of health care professionals. Drug resistance, drug side effects, wastage of resources, and serious health hazards including death are associated with self-medication. We conducted this study to find out the prevalence of self-medication among the peri-urban population of two randomly selected communities of Dharan, Nepal.

**Methods:**

A descriptive cross-sectional study was conducted among people residing in two randomly selected wards of peri-urban areas of Dharan from November 2017 and April 2018 after obtaining ethical clearance (IRC/1030/017). A pretested, structured self-administered questionnaire was used for data collection. Data were collected and entered in Statistical Package for the Social Sciences version 11.5; point estimate at 95% Confidence Interval was calculated along with frequency and proportion for binary data.

**Results:**

Among 426 respondents, the overall prevalence of self-medication was 312 (73.23%) at 95% Confidence Interval (67.83-78.63%). It was more common among female 158 (78.60%). Common symptoms were headache 201 (64.42%), fever 135 (43.26%), gastrointestinal 93 (29.8%) and respiratory illness 87 (27.88%). Analgesics and antipyretics 275 (88.14%) were the most common drugs selfmedicated with. Seeking opinion from pharmacist 112 (35.89%) was the commonest method adopted to procure drugs and comfort 127 (40.7%) and time constraints 122 (39.1%) were the commonest reasons.

**Conclusions:**

Prevalence of self-medication among the peri-urban population was similar to other studies. Headache and fever was the common symptoms for which self-medication were adopted. Awareness regarding potential dangers of self-medication and different drug side effects are recommended at the community level.

## INTRODUCTION

Self-medication practice (SMP) is the use of medication without prior medical consultation, guidance, or supervision of health care professionals.^1^ It is evolving as a reliable practice among people as it helps to treat minor illness, saves pressure, money, and time.^2^ In many developing countries where health care resources are still inadequate and many drugs are made available over the counter (OTC), it has become a boom.

However, the increasing prevalence of self-medication ranging from 0.1 to 100% is a matter of concern.^3^ If not practiced safely, dangerous drug interactions, development of drug-resistant pathogens and adverse drug reactions, wastage of resources, risk of dependence, and abuse can result from self-medication.

This study aims to find out the prevalence of selfmedication among the general population of randomly selected peri-urban wards of Dharan.

## METHODS

A descriptive cross-sectional study was conducted at the two randomly selected wards of peri-urban areas of Dharan from November 2017 to April 2018. Ethical approval was obtained from the Institutional Review Committee of B.P. Koirala Institute of Health Sciences (IRC/1030/017). Before the study, respondents were informed about the objectives of the study, and written consent was obtained.

The sample size was calculated by using the formula,

$$\begin{array}{ll} \text{n} & \text{= }{\text{Z}}^{\text{2}}\times \text{p }\times \text{(1}-\text{p)}/{\text{d}}^{\text{2}}\\ & \text{= }{(\text{1.96)}}^{\text{2}}\times \text{0.59}\times (1-0.59)/{(\text{0.05)}}^{\text{2}}\\ & \text{= }371.71\\ & =\;372 \end{array}$$

Where,

n = sample size
Z = 1.96 at 95% Confidence Interval
p = prevalence of self-medication, 59%^4^
e = margin of error, 5%

After adding a 10% non-response rate, the final sample size was 409. However, the study was done among 426 participants. By using a simple random sampling, two wards out of five peri-urban wards in the Dharan sub-metropolitan city were selected. From these two wards simple random sampling method was again used to select the houses. Heads of the households with age more than 18 years and willing to participate in the research were included in the study. Respondents with psychiatric illness or long-standing medical condition, pregnant females were excluded from the study. The confidentiality of the participants was maintained.

A structured, pretested questionnaire-based interview technique was used to conduct this survey, which was finalized after pretesting on 50 respondents and amended accordingly. The questionnaire mainly focused on symptoms and reasons of self-medication, category of drugs self-medicated with, sources of information, and the most common groups of drugs self-medicated with. Any use of over the counter (OTC) or prescription medicines without first consulting a doctor was considered as self-medication.

Data were analyzed using the Statistical Package of the Social Sciences version 11.5. For descriptive statistics, percentage mean, the standard deviation was calculated.

## RESULTS

Out of 426 respondents, 225 (52.81 %) were male, 201 (47.18%) were female. The prevalence of selfmedication among the people residing in peri-urban wards of Dharan was 312 (73.23%) at 95% CI (67.83-78.63%). The practice of self-medication was more common among females 158 (78.60%) (Table 1).

**Table 1:** Sociodemographic characteristics of participants (n= 426).

Character	Total (%)	Practicing self-medication	Not practicing self-medication
**Gender**			
**Male**	225 (52.81)	154 (68.44)	71 (31.55)
**Female**	201 (47.18)	158 (78.60)	43 (21.39)
**Age**			
**18-30**	92 (21.59)	61 (66.30)	31 (33.69)
**31-49**	183 (42.95)	150 (81.96)	33 (18.03)
**50-69**	87 (20.42)	53 (60.91)	34 (39.08)
**>70**	64 (15.02)	48 (75)	16 (25)
**Education**			
**No formal education**	115 (26.99)	86 (74.78)	29 (25.21)
**Primary school**	110 (25.82)	95 (86.36)	15 (13.63)
**Secondary school**	118 (27.69)	88 (74.57)	30 (25.42)
**Graduation or above**	83 (19.48)	43 (51.80)	40 (48.19)
**Marital status**			
**Unmarried**	67 (15.72)	46 (68.65)	21 (31.34)
**Married**	289 (67.84)	218 (75.43)	71 (24.56)
**Divorced**	42 (9.85)	27 (64.28)	15 (35.71)
**Widow(er)**	289 (67.84)	21 (75)	7 (25)
**Work status**			
**Unemployed**	72 (16.9)	46 (63.88)	26 (36.11)
**Employed**	207 (48.59)	168 (81.15)	39 (18.84)
**Self-employed**	94 (22.06)	61 (64.89)	33 (35.10)
**Retired**	53 (12.44)	37 (69.81)	16 (30.18)

The most common symptom demanding selfmedication was headache 201 (64.42%) and fever 135 (43.26%), followed by gastrointestinal complaints 93 (29.8%), respiratory symptoms 87 (27.88%) (Table 2) . Analgesics and antipyretics 275 (88.14%) were the most common drugs self-medicated with.

**Table 2:** Symptoms leading to self-medication (n = 426).

Symptoms	n (%)
Headache	201 (64.42)
Fever	135 (43.26)
Gastrointestinal complaints	93 (29.8)
Respiratory symptoms	87 (27.88)
Dysmenorrhea	15 (4.80)
Joint pain	53 (16.98)
Allergy	11 (3.52)

**Figure 1.**
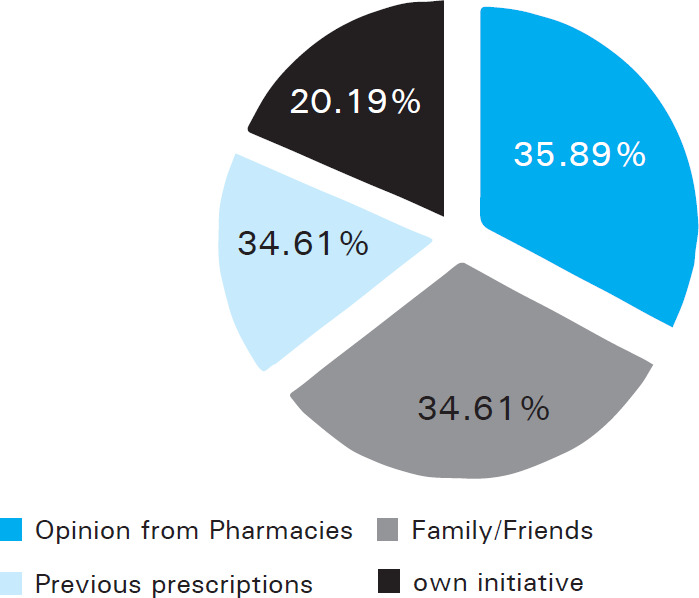
Source of information for self-medication.

Seeking opinion from pharmacist 112 (35.89%) was the commonest method adopted to procure drugs along with the use of the previous prescription with or without alteration of the dosage of the prescribed medications and sharing of drugs with family members and friends/ social circle (Figure 1).

**Figure 2.**
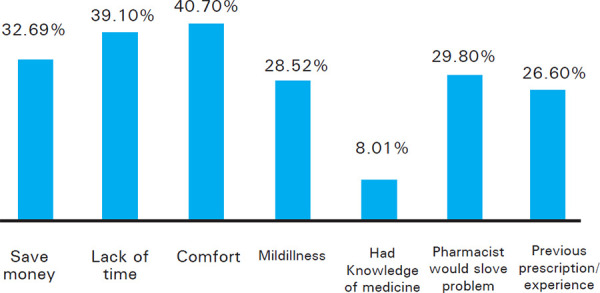
Reasons for self-medication.

Comfort 127 (40.7%) and time constraints 122 (39.1%) were the commonest reasons demanding the practice of self-medication (Figure 2).

## DISCUSSION

The findings of this study showed that the prevalence of self-medication was 73.23% among the peri-urban population of Dharan. This prevalence is considerably high, however in line with 76% prevalence rate reported among medical students in Karachi, Pakistan,^5^ 75% among medical and pharmacy students^6^ and 78.18% dentists^7^ in studies conducted in India. However, it is higher than the prevalence reported among the general public in Indonesia (45%),^8^ India (51.7%),^9^ Northeast Ethiopia,^10^ rural and urban population in Islamabad, Pakistan (61.20%)" and Italy (69.20%).^12^ These differences in the prevalence of SMP may be due to different sample sizes and the population and varying socio-demography. In the study conducted in Pakistan^11^ the sample size was 500 and the health care professionals found in the community were excluded from the study. The high degree of prevalence of selfmedication practice in our study could be due to the inadequate knowledge and information regarding the mishaps when not practiced safely. Also, the easy availability and accessibility of a wide range of drugs over the counter could be another contributing factor.

This study reports 68.44% prevalence of selfmedication among males and 78.60% among females which is inconsistent with the study reports from Italy^12^ where predominantly female (75.9%) practiced self-medication. Study reports from Nigeria^13,14^ also revealed females were more frequently involved in self-medication as compared to males. The majority (81.96%) of the respondents involved in SMP were 3149 years of age which is inconsistent with the findings of the study conducted in Nigeria,^13^ where middle-aged groups were more frequently involved in SMP. This may be because this age group is the full phase working for age group and are preoccupied with added responsibilities to be economically stable. This tends them to opt for an easy option to treat their illness without much time and financial loss.

More than two-thirds of the employed participants were more frequently involved in SMP in this study. This may be because visiting a doctor/hospital could be time-consuming. The main reason for getting involved in SMP was convenience/comfort (40.70%) and time limitation (39.10%). Other studies also report that the time factor is an important reason for getting involved in self-medication.^2,14,15^ The consultation charges of the doctor and the waiting time in the hospital costs both time and money, thus depending on the pharmacist (local chemist) was considered convenient. Also, opinion from the pharmacy/ chemist was the most common (35.89%) source of information, followed by family and friends (34.61%). Studies conducted by Mark, et al.^16^ and Ahmed et al. also revealed the same.^17^ The most common symptoms for which SM was practiced in this study were headache (64.42%), fever (43.36%), gastrointestinal symptoms (29.8%), respiratory symptoms like cough, cold (27.88%). Other studies conducted in India also reported these to be the frequent health complaints which were usually perceived as minor illness and demanded no experts' opinion.^18,19^ Almost half of the respondents (50.70%) stated they visited any health professionals only when sick and that was long back, 38.73% had their last visit more than 3 months back and 21.59% visited 1-3 months back. The category of medication mostly used in SMP in this study was analgesics/ antipyretics (88.14%) followed by antibiotics (22.11%) and drugs related to GI disorders (31.08%). Other studies also reported analgesics/antipyretics as the most common category of drugs self-medicated with.^20^

Participation of only head of the household was included in the study. Thus the result of this study may not be generalizable with the whole population. The data is self-reported depending on the respondent's honesty and ability to recall, this may be subject to recall bias.

## CONCLUSIONS

The prevalence of self-medication among the peri-urban population was high as compared to other studies. Headache and fever were the most common symptoms for self-medication followed by gastrointestinal and respiratory symptoms Analgesics and antipyretics were the most common drugs self-medicated with. Nearby pharmacies were the most common source for drug procurement. Preventive measures such as proper education and awareness programs regarding the potential harm of self-medication are further recommended.
